# The TWEAK-Fn14 System: Breaking the Silence of Cytokine-Induced Skeletal Muscle Wasting

**DOI:** 10.2174/156652412798376107

**Published:** 2012-01

**Authors:** S Bhatnagar, A Kumar

**Affiliations:** Department of Anatomical Sciences and Neurobiology, University of Louisville School of Medicine, Louisville, KY 40202, USA

**Keywords:** Denervation, disuse, inflammation, MuRF1, NF-kappa B, skeletal muscle, TWEAK.

## Abstract

The occurrence of skeletal muscle atrophy, a devastating complication of a large number of disease states and inactivity/disuse conditions, provides a never ending quest to identify novel targets for its therapy. Proinflammatory cytokines are considered the mediators of muscle wasting in chronic diseases; however, their role in disuse atrophy has just begun to be elucidated. An inflammatory cytokine, tumor necrosis factor (TNF)-like weak inducer of apoptosis (TWEAK), has recently been identified as a potent inducer of skeletal muscle wasting. TWEAK activates various proteolytic pathways and stimulates the degradation of myofibril protein both *in vitro* and *in vivo*. Moreover, TWEAK mediates the loss of skeletal muscle mass and function in response to denervation, a model of disuse atrophy. Adult skeletal muscle express very low to minimal levels of TWEAK receptor, Fn14. Specific catabolic conditions such as denervation, immobilization, or unloading rapidly increase the expression of Fn14 in skeletal muscle which in turn stimulates the TWEAK activation of various catabolic pathways leading to muscle atrophy. In this article, we have discussed the emerging roles and the mechanisms of action of TWEAK-Fn14 system in skeletal muscle with particular reference to different models of muscle atrophy and injury and its potential to be used as a therapeutic target for prevention of muscle loss.

## INTRODUCTION

Skeletal muscle wasting/atrophy is the major human morbidity observed during aging, a wide variety of chronic disease states (e.g. diabetes, chronic obstructive pulmonary disease, heart failure, renal failure, and cancer), disuse conditions (e.g. immobilization, denervation and unloading), and high dosage glucocorticoid therapy [1, 2]. Skeletal muscle wasting results from either enhanced protein degradation or reduced synthesis or both [1, 3, 4]. Diverse physiological and pathophysiological stimuli trigger muscle atrophy through the activation of specific intracellular signaling pathways and proteolytic systems. Generally, the insulin growth factor-1 (IGF-1)/insulin/ phosphatidylinositol 3-kinase (PI3K)/Akt pathway is considered as an anabolic pathway leading to increased protein synthesis and skeletal muscle hypertrophy [5-7]. On the other hand, activation of specific signaling proteins such as c-Jun-N-terminal kinase (JNK), p38 mitogen-activated protein kinase (MAPK), and AMP-activated protein kinase (AMPK) and/or transcription factors such as nuclear factor-κB (NF-κB), activator protein-1, p53, and Foxo lead to the loss of skeletal muscle mass through augmenting the activity of ubiquitin-proteasome system (UPS), autophagy, and caspases [8-19]. 

Whereas remarkable progress has been made towards understanding the intracellular pathways, little is known about the triggers and/or initial events responsible for loss of skeletal muscle mass in various catabolic states. Inflammatory cytokines have been suggested to induce and mediate local catabolic mechanisms at advanced stages of chronic diseases leading to the syndrome of cachexia [12, 20, 21]. However, their role in muscle atrophy in disuse conditions has just begun to be unfolded. Recent investigations have led to the identification of TWEAK-Fn14 system as a major regulator of skeletal muscle mass in both physiological and pathophysiological conditions. TWEAK appears to be the first cytokine involved in muscle wasting in disuse conditions. In this article, we have reviewed the current knowledge and discussed the mechanisms by which TWEAK-Fn14 system regulates skeletal muscle remodeling in different conditions. 

## OVERVIEW OF TWEAK-FN14 SYSTEM

TWEAK is a type II transmembrane protein and member 12 of the TNF super family (TNFSF) [22]. TWEAK is initially synthesized as a type II transmembrane protein, cleaved to its soluble form, and signals as a trimerized molecule [22, 23]. Both membrane-bound and soluble TWEAK proteins are fully functional though what regulates their relative abundance in specific conditions remains unknown. Fibroblast growth factor-inducible 14 (Fn14), a type I transmembrane protein was first recognized by differential display technique and later identified as the TWEAK receptor [24-26]. Fn14 is the smallest member of TNF receptor super family (TNFRSF). Its cytoplasmic domain contains a TNF receptor-associated factor (TRAF) binding site that allows recruitment of various TRAFs, which are also involved in cell signaling by other members of TNFSF [27]. One unique aspect of Fn14 not shared by other members of TNFRSF is that the expression of Fn14 is highly modulated under the influence of a wide variety of stimuli and conditions [23]. TWEAK-Fn14 dyad regulates several physiological responses including cell survival, proliferation, angiogenesis, migration, and apoptosis [23]. In contrast, increased expression of TWEAK and/or Fn14 is linked to pathogenesis in rheumatoid arthritis, systemic lupus erythematosus, multiple sclerosis, renal injury, stroke, neuroinflammation and neurodegeneration, several types of cancer, and cardiac dysfunction and failure [23, 28]. 

**Fig. (1):**
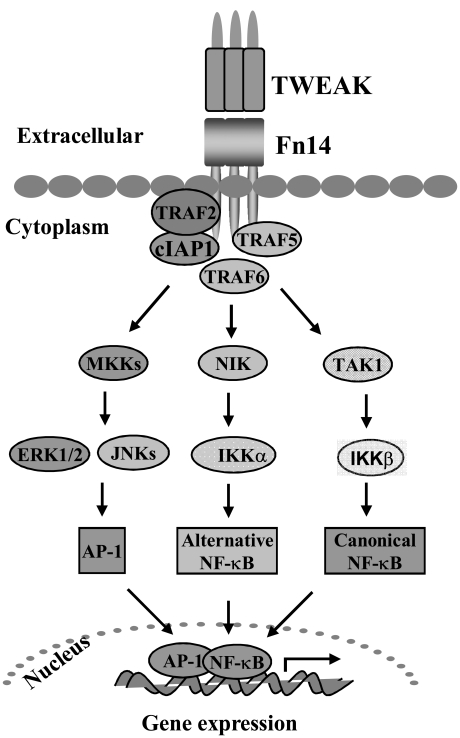
**The TWEAK-Fn14 signaling system.** Binding of TWEAK to Fn14 receptors leads to the recruitment of cIAP1, TRAF-2, 5, and/or 6 proteins leading to downstream activation of transforming growth factor β-activated kinase 1 (TAK1), NF-κB inducing kinase (NIK), and various mitogen-activated protein kinase kinases (MKK). Activation of TAK1 stimulates IκB kinase β (IKKβ) leading to the activation of canonical NF-κB. NIK phosphorylates and activates IKKα leading to downstream activation of NF-κB through alternative pathway. Various MKKs activate c-Jun N-terminal kinase 1 (JNK1) and p38 MAPK, which in turn activate transcription factor activator protein-1 (AP-1). Increased activation of NF-κB and AP-1 leads to the expression of specific genes resulting in TWEAK-mediated responses.

Like many other TNFSF members, TWEAK-Fn14 signaling mediates unique and context-dependent pleiotropic effects. For example, in contrast to TNF-α, TWEAK attenuates the transition from innate to adaptive immunity by suppressing the production of interferon-γ and IL-12 cytokines [29]. TWEAK has been shown to activate p44/p42 MAPK, c-Jun N-terminal kinase (JNK), transcription factor activator protein-1 (AP-1), and NF-κB signaling pathway in various cell types including skeletal muscle [30-33]. It has been suggested that binding of TWEAK to Fn14 extracellular domain leads to receptor trimerization, association of TRAF2/cIAP1 (cellular inhibitor of apoptosis protein 1) complex to cytoplasmic domain, and subsequent activation of various signaling proteins including TRAF6, transforming growth factor-β activated kinase1 (TAK1), I kappa B kinase (IKK), and MAPKs leading to altered expression of several genes involved in various cellular responses (Fig. **1**) [23, 30, 34-36]. TWEAK and Fn14 appear to have a minimal role in embryonic development or postnatal growth because mice null for TWEAK or Fn14 are viable and show no major abnormalities [29, 37, 38].

## TWEAK-Fn14 SYSTEM AND MUSCLE ATROPHY

Although classical inflammatory cytokines are known to cause muscle wasting, the limited success of anti-TNF-α or anti-IL-1β therapy in prevention of muscle loss has suggested that there might be other mediators of skeletal muscle wasting [39, 40]. Using C2C12 myotubes as a model system, we previously evaluated the effects of several members of TNFSF on myotube size. These initial experiments revealed that treatment with physiological concentrations of TWEAK causes significant reduction in myotube diameter [41]. Muscle wasting involves the degradation of selective muscle proteins and myosin heavy chain (MyHC) is one such protein that undergoes rapid proteolysis in different atrophic conditions such as denervation, starvation, tumor load, and in response to inflammatory cytokines [34, 42, 43]. Consistent with its catabolic role in myotubes, TWEAK was found to reduce the levels of MyHC in cultured myotubes in a dose-dependent manner [41]. 

The UPS is a major proteolytic system that causes selective degradation of regulatory and structural proteins in skeletal muscle [44, 45]. Almost a decade back, two muscle-specific E3 ubiquitin ligases, muscle RING-finger 1 (MuRF1) and muscle atrophy F-box (MAFbx; also known as Atrogin-1) were identified [46, 47]. The expression of MuRF1 and MAFbx has been found to be increased in multiple models of skeletal muscle atrophy [46-48]. Catabolic role of these ubiquitin ligases has been validated by the findings that muscle wasting is considerably inhibited in mice null for MuRF1 or MAFbx compared to wild-type in response to a wide variety of stimuli [46-48]. Accumulating evidence also suggests that MuRF1 targets specific thick filament proteins including MyHC for proteolysis in skeletal muscle [49, 50].

Cell culture studies showed that the treatment with TWEAK increases the expression of muscle-specific E3 ubiquitin ligases MuRF1 and MAFbx and stimulates the conjugation of ubiquitin with MyHC in C2C12 myotubes suggesting that TWEAK causes degradation of MyHC through the activation of UPS [41]. More recently, we have reported that TWEAK can also induce the expression of components of the autophagy-lysosomal system and activates caspases especially caspase-3 in cultured myotubes, which may also contribute to myofibrillar proteolysis [51]. Furthermore, TWEAK inhibits the activity of PI3K/Akt signaling pathway which further explains the strong catabolic actions of TWEAK in cultured myotubes [41]. 

The *in vivo* effects of TWEAK on skeletal muscle were investigated by chronic administration of soluble TWEAK protein in wild-type mice and through generation of muscle-specific TWEAK-transgenic (Tg) mice. Treatment of mice with TWEAK led to a significant reduction in body weight and individual hind limb muscle weight and fiber-cross-sectional area compared to untreated littermates [41]. Furthermore, transgenic overexpression of full-length TWEAK cDNA using muscle creatine kinase promoter showed profound loss of skeletal muscle mass and neonatal lethality (in high copy number transgenic founder) in mice [41]. Founder TWEAK-Tg mice were smaller in size and had difficulty in breathing and motion. TWEAK-Tg mice which survived due to relatively low expression of TWEAK (4-6 folds higher than littermate wild-type mice) showed atrophy and interstitial fibrosis around six months of age [34]. Consistent with *in vitro* studies, the activation of NF-κB, levels of MuRF1, and ubiquitination of MyHC were significantly elevated in skeletal muscle of TWEAK-treated or TWEAK-Tg mice compared to their corresponding controls indicating that TWEAK causes muscle wasting through activation of NF-κB and enhancing the expression of the components of UPS especially MuRF1 [41]. Interestingly, while TWEAK induced the expression of MAFbx in cultured myotubes [41], there was no significant difference in mRNA levels of MAFbx in skeletal muscle of wild-type and TWEAK-Tg mice indicating that TWEAK might be causing muscle-wasting *in vivo* specifically by augmenting the expression of MuRF1 [41]. 

The role of TWEAK-Fn14 system in physiological atrophy was investigated through a series of experiments in our laboratory. Gene array experiments showed that the expression of TWEAK receptor Fn14 is regulated in the conditions of atrophy and hypertrophy [34]. Fn14 levels were found to be increased in skeletal muscle in various disuse conditions such as denervation and immobilization [34]. Recently, Wu *et al.* [52] studied the global gene expression in skeletal muscle of mice in response to hind-limb suspension, a model of unloading-induced skeletal muscle atrophy. This study identified a number of genes upregulated after 6 days of hind limb suspension [52]. Interestingly, both microarray and quantitative real-time PCR assays, showed that the expression of Fn14 is significantly increased in gastrocnemius muscle of mice at 6 days of hind limb suspension further supporting the inference that the expression of Fn14 goes up in various disuse conditions [52]. Conversely, hypertrophy stimuli such as recovery after cast immobilization or exercise reduced even the basal level of Fn14 in skeletal muscle [34]. It is noteworthy that TWEAK-Fn14 system is not involved in all types of muscular atrophy. High dose of glucocorticoids*,* which causes severe muscle-wasting, did not affect the levels of TWEAK or Fn14 in skeletal muscle of mice [34]. Similarly, the expression of either TWEAK or Fn14 did not change in response to inflammatory cytokines and endotoxin in cultured myotubes (our unpublished observations) further suggesting that TWEAK-Fn14 might be causing skeletal muscle wasting in only specific conditions. 

To evaluate the role of TWEAK in disuse atrophy, hind limb muscle of wild-type, TWEAK-Tg, and TWEAK-KO mice were denervated (meaning transection of sciatic nerve) for 10-12 days. Interestingly, skeletal muscle mass and functions were considerably preserved in TWEAK-KO mice compared to age-matched wild-type mice upon denervation [34]. In contrast, the denervation-induced muscle atrophy and fibrosis were significantly increased in TWEAK-Tg mice compared to wild-type littermates [34]. Furthermore, pharmacological inhibition of TWEAK using an TWEAK neutralizing antibody also rescued the denervation-induced muscle atrophy in wild-type mice [34]. TWEAK was found to stimulate the activation of NF-κB and the expression of MuRF1 (but not MAFbx) in denervated skeletal muscle [34, 41]. Coincidently, this was the first report providing experimental evidence about the involvement of an inflammatory cytokine in skeletal muscle in disuse/denervation conditions [34]. All previous attempts to investigating the role of inflammatory cytokine in disuse atrophy were focused on classical muscle-wasting cytokines such as TNF-α, IL-1β, IL-6, and IFN-γ. Gene expression studies found no evidence for their involvement in disuse-related muscle atrophy [1, 53, 54].

**Fig. (2):**
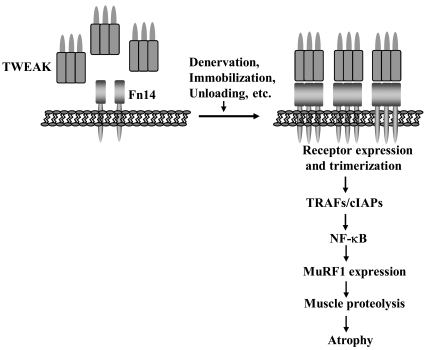
**Mechanisms of action of TWEAK in disuse atrophy.** In normal healthy skeletal muscle, Fn14 expression is relatively low but it is drastically increased in various disuse conditions. Overexpression of Fn14 may promote its trimerization as is the case with many other members of TNF receptor super family. Binding of TWEAK to Fn14 results in activation of NF-κB leading to increased expression of MuRF1 and atrophy.

It was interesting to note that TWEAK did not affect all pathways known to be involved in regulation of skeletal muscle mass. While TWEAK inhibits Akt phosphorylation in cultured myotubes [41], the level of phosphorylation of Akt and its downstream targets was comparable in denervated muscle of wild-type, TWEAK-Tg and TWEAK-KO mice suggesting that the inhibitory effect of TWEAK on PI3K/Akt pathway is neutralized by some other factors that may be present in denervated muscle *in vivo* [34]. Furthermore, a significant increase in the expressions of several autophagy-related genes (e.g. LC3B, Beclin1, Atg5, Atg12, and Gabarapl1) was also noticeable in skeletal muscle upon denervation. However, there was no significant difference in mRNA levels of various autophagy-related genes between denervated muscle of wild-type, TWEAK-Tg, and TWEAK-KO mice [34]. These studies thus indicate that TWEAK-Fn14 dyad specifically up-regulates the expression of the components of the UPS in denervated muscle. Similar mechanisms might be involved in other disuse conditions such as immobilization and unloading (Fig. **2**). 

While it is now clear that the expression of Fn14 is a rate-limiting step in TWEAK-mediated muscle wasting, the underpinning mechanisms by which Fn14 is up-regulated in skeletal muscle in conditions of atrophy have not been yet investigated. Previous *in silico* analyses of the promoter regions of both human and mouse Fn14 gene had suggested the presence of consensus binding sites for a number of transcription factors including NF-κB, AP-1, SP-1, and MyoD [55]. Recently, Wu *et al.* reported that unloading-induced upregulation of Fn14 is significantly inhibited in *Nfkb1*-knockout mice supporting role of NF-κB in the increased expression of Fn14 in disuse conditions [52]. Furthermore, Fn14 gene appears to contain CpG rich regions in its promoter (our unpublished observations) indicating that the expression of Fn14 may also be regulated through epigenetic mechanisms. 

In addition to activating proteolysis, TWEAK also up-regulates the expression of matrix metalloproteinases (MMPs), especially MMP-9, which may be responsible for extracellular matrix breakdown and fibrosis in atrophying skeletal muscle [30]. TWEAK also modulates the expression of a number of genes and microRNAs (miRs) known to be involved in manifestation of oxidative stress, mitochondrial abnormalities, fibrosis, and energy deficit in skeletal muscle [56, 57]. The precise role of specific genes and miRs affected by TWEAK in skeletal muscle is an important area for future investigations. 

## TWEAK VERSUS OTHER CATABOLIC CYTOKINES

Although comparative studies are still lacking, there appears to be some overlap as well as distinction between the mechanisms of action of TWEAK and other inflammatory cytokines such as TNF-α, IL-1β, and IL-6 in skeletal muscle wasting. This contention is also supported by our recent microarray studies which showed that TNF-α and TWEAK affect the expression of a large number of common and distinct sets of genes and molecular pathways [56, 58]. A recent study employing cultured myotubes has suggested that TNF-α acts by stimulating FOXO4 directly and not through Akt kinase which ultimately modulates FOXO1/3 signaling [59]. While TNF-α has been reported to augment the phosphorylation of Akt in cultured myotubes [59], TWEAK inhibits even the basal level of phosphorylation of Akt kinase in myotubes suggesting distinct modes of action of these catabolic cytokines [41]. Li *et al. *[60] have previously reported that TNF-α also stimulates the release of reactive oxygen species and the activation of p38 MAPK, which stimulates the expression of MAFbx. Additionally, activation/ expression of MuRF1 has been proposed as a mechanism by which TNF-α down-regulates troponin T leading to loss of skeletal muscle function [61]. Although TWEAK induces the expression of MAFbx and MuRF1 [41] and the activation of p38MAPK in C2C12 myotubes [57], it remains to be investigated whether TWEAK also induces the expression of MAFbx or MuRF1 through augmenting oxidative stress and/or activation of p38MAPK. Similar to TNF-α and TWEAK, a recent study by Li* et al.* [62] has demonstrated that both IL-1α and IL-1β cause significant atrophy when added to cultured myotubes. However, IL-1 acts through an oxidant- and Akt/Foxo-independent mechanism to activate p38 MAPK and stimulate NF-κB leading to enhanced expression of MAFbx and MuRF1 and myofibrillar proteolysis in C2C12 myotubes [62]. 

One of the unique features of TWEAK that is not shared by TNF-α or other muscle-wasting cytokines is its effect on NF-κB signaling pathway. Depending on the type of stimuli, the activation of NF-κB can occur *via *either a canonical or alternative pathway [63]. The canonical NF-κB signaling pathway involves the upstream activation of inhibitors of κB (IκB) kinase-β (IKKβ) and subsequent phosphorylation and degradation of IκB proteins [63]. On the other hand, activation of the alternative NF-κB pathway requires the upstream activation of NF-κB-inducing kinase (NIK) and IKKα and the proteolytic processing of p100 subunit into p52 [63]. TNF-α predominately activates the canonical NF-κB signaling pathway [12] though there is also now evidence that continued presence of TNF-α may also activate alternative NF-κB pathway to some extent in myotubes [58]. By contrast, TWEAK is a potent activator of both canonical and alternative NF-κB signaling pathway in skeletal muscle [57]. Furthermore, while the effect of TNF-α on NF-κB activation is rapid and transient, TWEAK causes slow but sustained activation of NF-κB in both myoblasts and myotubes [41, 64]. We believe that the stronger effects of TWEAK compared to TNF-α on MyHC degradation in cultured myotubes [41], is attributed, at least in parts, to the TWEAK’s ability to cause sustained activation of NF-κB, which stimulates the activity of UPS through augmenting the expression of MuRF1 and other components of proteasome [9, 65]. It is also noteworthy that the overexpression of a degradation-resistant mutant of IκBα (i.e. IκBα∆N) preserves the levels of MyHC in C2C12 myotubes in response to both TNF-α and TWEAK suggesting that the activation of classical NF-κB pathway predominately mediates the degradation of MyHC in response to both these cytokines [41, 66]. While the exact role of alternative NF-κB signaling pathway in muscle wasting remains to be investigated, a recent study suggested that the alternative NF-κB signaling pathway promotes mitochondrial biogenesis in differentiated myotubes [67]. However, in this study, the role of alternative pathway was studied in unchallenged myotubes [67]. The exact role of alternative pathway may depend on many factors including the type of stimuli and the length and level of activation of NF-κB. 

IL-6 also causes loss of muscle mass; however, it appears that protein synthesis is suppressed more than proteolysis [68] which could be attributed to a reduction in Akt activity [69]. With regard to muscle proteolysis, circulating concentrations of IL-6 in the cachectic APC^min/+^ mouse have been shown to increase MAFbx level and cause atrophy of type IIB fibers [70]. Other evidence suggests IL-6 may not increase MAFbx or MuRF1 [71]. IL-6 is increased in chronic kidney disease. This increase in IL-6 along with serum amyloid A impairs insulin/IGF-I signaling as indicated by Akt activity and results in muscle proteolysis [69]. 

## TWEAK AND FIBER-TYPE COMPOSITION

Skeletal muscle of adult mouse contains four types of fibers: I, IIA, IIX, and IIB based on the MyHC isoform that is primarily expressed [72, 73]. MyHC type I fibers are “slow oxidative” that have high mitochondrial content, favor fatty acid oxidation, and have slower contraction/relaxation profile. MyHC type II fibers are called “fast glycolytic” fibers. Type IIA and type IIX fibers are fast oxidative/glycolytic fibers that have intermediate biophysical properties but tend to be oxidative too and rich in mitochondria [72, 73]. Adult skeletal muscle has enormous plasticity [74]. For example, endurance training induces fast-to-slow fiber type switch leading to improved endurance performance and resistance to fatigue. In contrast, resistance exercise induces hypertrophy, a slow-to-fast-fiber transformation, and a switch to induce glycolysis as the favored energy source [74]. Interestingly, disuse and cancer cachexia cause not only atrophy but also induce slow-to-fast type fiber switch. Furthermore, different fiber-types display different sensitivity to atrophy; oxidative fibers are somewhat resistant to atrophy upon denervation [75] whereas glycolytic fibers undergo atrophy at a faster rate during starvation or sepsis than oxidative fibers [76, 77]. 

Analyses of fiber-type composition in skeletal muscle of TWEAK-Tg and TWEAK-KO mice provided interesting results. Transgenic overexpression of TWEAK led to a significant reduction in type I fibers with a concomitant increase in type II fibers in both soleus and extensor digitorum longus (EDL) muscle [34]. Furthermore, hind limb muscle of the founder TWEAK-Tg mice which did not survive beyond neonatal age, appeared pale compared to more reddish muscle of wild-type littermates [41]. Moreover, compared to wild-type mice, an increased proportion of type I fibers was observed in skeletal muscle of TWEAK-KO [34] suggesting that TWEAK favors fast-type fiber phenotype even in unchallenged skeletal muscle.

The mechanisms by which TWEAK causes slow-to-fast-type fiber transformation have not been yet investigated. Peroxisome proliferator-activated receptor-gamma (PPAR-γ) coactivator 1α (PGC-1α) is a key player in regulating skeletal muscle fiber composition, mitochondrial content, and oxidative metabolism in both physiological and pathophysiological conditions [78-80]. Transgenic mice expressing PGC-1α in skeletal muscle have increased proportion of fibers expressing MYHC I and IIA [81]. These effects of PGC-1α are mediated, at least in part, through augmenting the activity of MEF2 transcription factor, which induces type I fiber genes [82]. Interestingly, it was recently reported that TWEAK suppresses the expression of MEF2 in myofibers [56]. Accumulating evidence also indicates that PGC-1α preserves skeletal muscle mass in various catabolic states including denervation [83, 84]. There is a possibility that elevated levels of TWEAK increase the proportion of type II fibers through diminishing the expression of PGC-1α. The potential relationship between TWEAK-Fn14 system and PGC-1α is also suggested by the observations that the levels of PGC-1α in skeletal muscle go up after exercise [85-87]. In contrast, the levels of Fn14 are reduced below the basal level in exercised animals or in response to hypertrophy stimuli [34]. Since type II fibers undergo atrophy at faster rate compared to slow-type fibers, it is likely that TWEAK causes muscle loss by first inducing fiber-type transformation followed by the activation of various catabolic systems. Consistent with this hypothesis, recent studies have shown that TNF-α, another muscle-wasting cytokine, down regulates the expression of PGC-1α in cultured myotubes [88, 89]. Experiments are in progress in our laboratory to determine whether crossing of PGC-1α Tg with TWEAK-Tg mice can prevent the slow-to-fast-type fiber transformation and muscle atrophy in TWEAK-Tg mice. Furthermore, it will be interesting to investigate whether exercise capacity and mitochondrial content are improved in TWEAK-KO and reduced in TWEAK-Tg mice compared to wild-type littermates and if so whether this occurs through modulation of expression of PGC-1α. 

## TWEAK/Fn14 SIGNALING IN MYOGENESIS

Skeletal muscle formation or myogenesis is a complex and highly regulated process that involves the determination of multipotent mesodermal cells to generate myoblasts, exit of these myoblasts from the cell cycle, and their differentiation into muscle fibers [90, 91]. Myogenesis is regulated by the sequential expression of myogenic regulatory factors (MRFs), a group of basic helix-loop-helix transcription factors that include Myf5, MyoD, myogenin, and MRF4 [92]. During myogenesis, fusion of myoblasts into multinucleated myotubes is the terminal step of differentiation after which no further mitotic divisions occur within the myotubes or muscle fibers. The extra nuclei required for muscle growth are provided by satellite cells, which are located under the basal lamina of the muscle fiber [92]. 

While TWEAK cytokine is widely expressed by a number of cell types, its receptor Fn14 is expressed only in selective cell types [23]. Fluorescence activated cell sorting and biochemical assays showed that Fn14 is highly expressed in primary mouse myoblasts and C2C12 myoblastic cell line [33]. Cultured myoblasts respond to TWEAK by increased activation of various signaling pathways [33, 64]. Skeletal muscle of TWEAK-null or Fn14-null mice are normal [34, 37] suggesting that TWEAK and Fn14 are either not required or their deficiency is compensated by other factors present during muscle development. In contrast, increased levels of TWEAK affect both proliferation and differentiation of muscle progenitor cells. Addition of exogenous TWEAK protein augments proliferation but inhibits the differentiation of cultured myoblasts [64]. Analyses of TWEAK-treated cultures further showed that TWEAK reduces the levels of myogenin and MyoD in differentiating myoblasts [64]. While the levels of myogenin are diminished due to suppression of transcription, TWEAK stimulates the proteolytic degradation of MyoD in differentiating C2C12 cultures [64]. Furthermore, it was found that one of the mechanisms by which TWEAK inhibits myogenesis is through the activation of NF-κB. TWEAK causes sustained activation of NF-κB and the inhibition of NF-κB through overexpression of a kinase dead mutant of IKKβ (an upstream activator of NF-κB) or IκBαΔN (a degradation resistant mutant of NF-κB inhibitory protein IκBα) rescued myogenic differentiation in TWEAK-treated C2C12 myoblasts [64]. Moreover, NF-κB is responsible, at least in part, for the enhanced degradation of MyoD in TWEAK-treated myoblasts [64]. These effects of TWEAK on myoblasts are consistent with published reports suggesting that other inflammatory cytokines also inhibit myogenic differentiation through the activation of NF-κB and reducing the levels of MyoD [93, 94]. Activation of NF-κB through canonical pathway generally inhibits the differentiation of myoblasts into myotubes [12, 67, 95] whereas the activation of NF-κB through alternative pathway may have a positive role in mitochondrial biogenesis and maintenance of differentiated phenotype of myotubes [67].

Because TWEAK inhibits myogenic differentiation, it was hypothesized that the suppression of TWEAK receptor Fn14 in myoblasts would enhance their differentiation into myotubes. However, contrary to this presumption, knockdown of Fn14 by siRNA technique blocked the formation of myotubes in both C2C12 and mouse primary myoblast cultures [33]. Similar results were reported by Girgenrath *et al. *[37] where the authors showed that (a) Fn14 is highly expressed on muscle progenitor cells; (b) recombinant TWEAK protein inhibits myogenic differentiation by preventing cell cycle arrest in myoblasts; and (c) TWEAK suppressed the expression of both myogenin and MyoD in differentiating myoblasts [37]. Their study also showed that primary myoblasts from Fn14-null mice display significantly reduced proliferative capacity and altered myotube formation *in vitro* further validating a positive role of Fn14 in myogenesis [37]. These observations along with other published reports demonstrating that overexpression of Fn14 alone can induce various cellular responses including tumor cell migration and invasion [96-98] suggest that TWEAK-independent Fn14 signaling also occurs which causes distinct biological responses compared to the settings where TWEAK is also present. Indeed, knockdown of Fn14 impaired the activation of RhoA GTPase and serum response factor in differentiating myoblasts providing a possible mechanism by which Fn14 might be regulating myogenic differentiation [33]. There is also a possibility that in the absence of TWEAK, Fn14 predominantly activates promyogenic signaling pathways. Moreover, low levels of TWEAK may be required for proliferation of muscle progenitor cells. However, when TWEAK is present in sufficient amount to bind to Fn14, it leads to the activation of specific signaling pathways such as MAPKs and NF-κB which may counter the promyogenic effects of Fn14 by inhibiting the withdrawal of myoblasts from cell cycle [33].

## TWEAK/Fn14 SIGNALING IN SKELETAL MUSCLE REGENERATION

Skeletal muscle regeneration is a multi-step process that involves the participation of a number of autocrine and paracrine factors [90, 91, 99]. The inflammatory response which starts within hours of injury and lasts up to seven days involves accumulation of neutrophils and macrophages which help removing tissue debris through phagocytosis in injured skeletal muscle [100]. These infiltrating phagocytes in injured skeletal muscle are also known to produce a number of cytokines and chemokines which can affect the proliferation and differentiation of satellite cells [100]. While it is now clear that the recruitment of phagocytes is critical for efficient regeneration of injured muscle, the roles of various proinflammatory and anti-inflammatory cytokines in skeletal muscle regeneration has not been yet clearly elucidated. Cell culture studies have suggested that inflammatory cytokines can modulate both the proliferation and differentiation of myogenic cells, however, the direct correlation between *in vitro* and *in vivo* studies is still lacking. There may also be some redundancy among cytokines regarding their role in skeletal muscle regeneration. For example, while TNF-α strongly affects the survival, proliferation, and differentiation of cultured myoblasts [93, 94], skeletal muscle regeneration was normal in TNF-null mice [101]. 

**Fig. (3):**
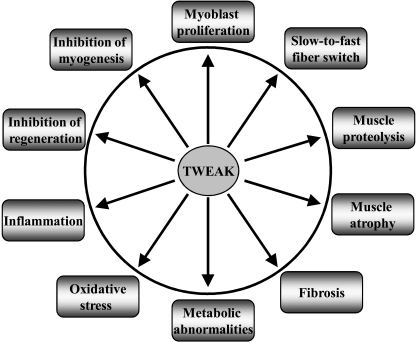
**TWEAK regulation of skeletal muscle biology.** TWEAK-induced affects on muscle progenitor cells and adult skeletal muscle that have been elucidated so far. ECM, extracellular matrix.

The *in vivo* role of TWEAK in skeletal muscle regeneration has now been investigated employing both TWEAK-KO and muscle-specific TWEAK-transgenic (Tg) mice [102]. The expression of both TWEAK and Fn14 is significantly elevated within 3-5 days following cardiotoxin (CTX) injection, a known trigger for satellite cell-driven skeletal muscle regeneration [102]. When muscle regeneration was evaluated, no obvious difference in muscle structure was observed between wild-type, TWEAK-KO, and TWEAK-Tg mice, 5 days after CTX injection. However, at 10 and 21 days post CTX-injection, regenerating myofibers of TWEAK-KO mice appeared larger in diameter compared to wild-type mice [102]. By contrast, regenerating fibers were smaller in size in TWEAK-Tg mice compared to wild-type littermates [102]. Further analyses of muscle using biochemical and histological techniques showed that TWEAK mediates the inflammatory response leading to diminished regeneration and/or growth. The levels of activation of NF-κB, expression of specific inflammation-related molecules, and interstitial fibrosis were significantly reduced in regenerating muscle of TWEAK-null mice and exacerbated in TWEAK-Tg mice compared to wild-type mice [102]. Although the exact mechanisms by which TWEAK regulates the size of regenerating myofibers is not clear, there are two possibilities which can account for the observed effects of TWEAK. First, it is possible that TWEAK by itself or in association with other inflammatory cytokines blocks the fusion of satellite cells resulting in reduced differentiation and growth of myofibers [64, 93]. Second, TWEAK may directly act on regenerating myofibers leading to the activation of various proteolytic systems and hence reduced fiber diameter [34, 41]. Together, these observations suggest that TWEAK inhibits the genesis and growth of skeletal muscle after injury. Reduced regeneration of myofibers after injury may be another determinant of TWEAK-induced skeletal muscle wasting (Fig. **3**).

It is interesting to note that the role of TWEAK and Fn14 in adult skeletal muscle regeneration is quite similar to their individual role in myogenic differentiation. Fn14-KO mice showed delayed muscle regeneration after injury [37]. Number of newly formed fibers with centronucleation and/or positive for embryonic form of myosin heavy chain was significantly reduced in Fn14-KO mice compared to wild-type mice in TA muscle at 5 and 7 days following cardiotoxin injection [37]. While muscle regeneration was almost complete by 14 days in wild-type mice, residual muscle damage along with regenerated fibers and inflammatory cells still persisted in Fn14-KO mice further suggesting fiber regeneration is diminished in Fn14-KO mice [37]. This study also suggested that the delayed muscle regeneration in Fn14-knockout mice could be attributed to diminished/delayed inflammatory response. Immune cells such as macrophages and neutrophils infiltrate muscle tissue within 1-3 days post CTX injection which helps removing tissue debris in injured muscle [100]. Concentration of both macrophages and neutrophils were significantly lower in Fn14-KO mice compared to wild-type mice measured 3 days post CTX injection [37]. Furthermore, it is likely that the delayed inflammatory response occurs due to suppression of expression of chemokine MCP-3 in Fn14-KO mice after CTX injection [37]. Certainly, more investigations are required to delineate the mechanisms by which TWEAK and Fn14 differentially regulate both myogenesis and adult skeletal muscle regeneration. 

## CONCLUDING REMARKS

The studies summarized above indicate that TWEAK-Fn14 system plays an important role in skeletal muscle remodeling. Most of these observations suggest that TWEAK-Fn14 signaling increases skeletal muscle loss and inhibition of this cytokine-receptor axis can be used as a therapy to preserving skeletal muscle mass in the conditions of atrophy and muscle injury. We believe that TWEAK-Fn14 system is among the most attractive drug targets for prevention of muscle wasting. Because TWEAK is an extracellular protein, TWEAK-dependent signaling can be blocked using a TWEAK neutralizing antibody or soluble Fn14-Fc decoy protein, which can prevent TWEAK binding to Fn14 cell surface receptors. Indeed, these two reagents have been found to improve pathology in animal models of some other diseases where TWEAK-Fn14 signaling is elevated. Alternatively, small-molecule antagonists that prevent Fn14 trimerization or interaction of TWEAK with Fn14 can also be used for blocking catabolic effects of TWEAK on skeletal muscle. 

Whereas the role of TWEAK-Fn14 signaling in skeletal muscle is increasingly clear, there are still many outstanding questions which need to be addressed. For example, it is important to identify other conditions in which TWEAK is a mediator of skeletal muscle atrophy. Since TWEAK inhibits the activation of PI3K/Akt pathway, it is likely that TWEAK might also be causing muscle atrophy in the settings of diabetes and starvation where drop in insulin levels and inhibition of PI3K/Akt pathway are the major mechanisms of muscle loss. The mechanisms by which TWEAK-Fn14 system causes muscle atrophy also require further investigation. While TWEAK has been found to induce the activation of NF-κB and MuRF1, this cannot explain all the affects that TWEAK produces in skeletal muscle. For example, the mechanisms by which TWEAK induces fiber-type transformation in skeletal muscle remains to be investigated. Future studies focusing to examine the effects of TWEAK on expression of PGC-1s and its associated transcription factors such as nuclear respiratory factor (NRF)-1 and NRF-2 may provide significant insight into the mechanisms of action of TWEAK leading to slow-to-fast-type fiber switch. It will also be interesting to examine whether TWEAK affects mitochondrial content by affecting the processes of mitochondrial biogenesis, fusion, and/or fission. TWEAK inhibits the expression of several micro RNAs (miRs) including muscle-specific miR1, miR133, and miR206, which could also be a potential mechanism by which TWEAK causes the loss of skeletal muscle mass in various catabolic conditions. 

## ABBREVIATIONS

CTX: = cardiotoxin
IKK: = I kappa B kinase
KO: = knockout
MAFbx: = muscle atrophy F-box
MAPK: = mitogen-activated protein kinase
MyHC: = myosin heavy chain
MuRF1: = muscle RING-finger 1
NF-κB: = nuclear factor-kappa B
PGC-1α: = peroxisome proliferator-activated receptor-gamma coactivator 1α
PI3K: = phosphatidylinositol 3-kinase
Tg: = transgenic
TNF: = tumor necrosis factor
TNFSF: = TNF super family
TWEAK: = TNF-like weak inducer of apoptosis
UPS: = ubiquitin-proteasome system
